# Coarse-grained simulation reveals key features of HIV-1 capsid self-assembly

**DOI:** 10.1038/ncomms11568

**Published:** 2016-05-13

**Authors:** John M. A. Grime, James F. Dama, Barbie K. Ganser-Pornillos, Cora L. Woodward, Grant J. Jensen, Mark Yeager, Gregory A. Voth

**Affiliations:** 1 Department of Chemistry, Institute for Biophysical Dynamics, James Franck Institute, and Computation Institute, The University of Chicago, Chicago, Illinois 60637, USA; 2 Department of Molecular Physiology and Biological Physics, University of Virginia School of Medicine, Charlottesville, Virginia 22908, USA; 3 Division of Biology, California Institute of Technology, 1200 E. California Blvd., Pasadena, California 91125, USA; 4 Howard Hughes Medical Institute, California Institute of Technology, 1200 E. California Blvd., Pasadena, California 91125, USA; 5 Center for Membrane Biology, Cardiovascular Research Center, and Division of Cardiovascular Medicine, University of Virginia School of Medicine, Charlottesville, Virginia 22908, USA

## Abstract

The maturation of HIV-1 viral particles is essential for viral infectivity. During maturation, many copies of the capsid protein (CA) self-assemble into a capsid shell to enclose the viral RNA. The mechanistic details of the initiation and early stages of capsid assembly remain to be delineated. We present coarse-grained simulations of capsid assembly under various conditions, considering not only capsid lattice self-assembly but also the potential disassembly of capsid upon delivery to the cytoplasm of a target cell. The effects of CA concentration, molecular crowding, and the conformational variability of CA are described, with results indicating that capsid nucleation and growth is a multi-stage process requiring well-defined metastable intermediates. Generation of the mature capsid lattice is sensitive to local conditions, with relatively subtle changes in CA concentration and molecular crowding influencing self-assembly and the ensemble of structural morphologies.

## Abstract (web summary)

Significant morphological changes occur during the conversion of the immature HIV virion into a mature infectious form. Here the authors use coarse-grained molecular dynamics simulations to model HIV-1 capsid self-assembly and disassembly events that suggests several metastable capsid intermediates sensitive to local conditions.

## Graphical abstract

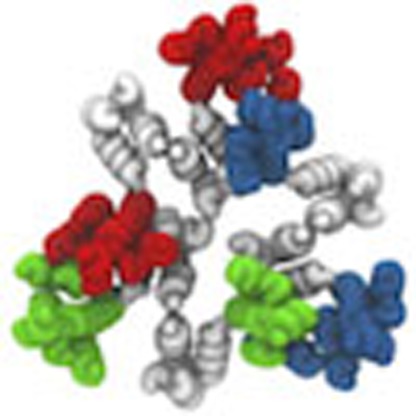

Significant morphological changes convert an ‘immature' virus particle (virion) of HIV-1 into the mature and infectious form^1^,^2^,^3^. During maturation, enzymatic cleavage of Gag polypeptide^4^ releases capsid protein (CA) to self-assemble into a conical lattice structure enclosing the viral RNA (the capsid, Fig. 1). Failure to generate a suitable capsid precludes infectivity^5^,^6^,^7^,^8^,^9^ and so the details of capsid generation are of significant interest as a therapeutic target.

HIV-1 capsid protein is mainly dimeric under physiological conditions, with CA monomers connected by a well-characterized interface between C-terminal domains (CTDs)^10^. Given suitable conditions *in vitro*, CA can spontaneously self-assemble into a wide variety of structures^11^,^12^,^13^,^14^,^15^,^16^,^17^,^18^, but the prototypical mature virion contains a single conical capsid with a complex of viral RNA and nucleocapsid protein (NC) condensed within the broader terminus. In agreement with fullerene cone models^14^, pentameric and hexameric CA oligomers were identified as the basic components of mature-style CA lattice, with CA N-terminal domains (NTDs) arranged into quasi-equivalent rings^15^,^19^,^20^,^21^, and with pentamers believed to be located in regions of higher lattice curvature^21^,^22^. Although the NTD and CTD structures of CA are well conserved, inter-domain flexibility allows CA in solution to dynamically transition between conformations that are compatible and incompatible with the mature lattice^23^.

Controlled study of HIV-1 capsid assembly is complicated by the inherent variability of virions: non-trivial differences in average radius (≈630±50 Å) and Gag content (≈2,400±700 molecules) are reported *in vivo*^24^,^25^,^26^, and these values bound a significant potential range of CA concentrations. The large numbers of Gag, in addition to viral RNA and sundry molecules sequestered from the host cell^27^,^28^, ensure the virion interior is a highly crowded environment with internal organic mass densities as high as ≈300 mg ml^−1^ (ref. 24). Molecular-crowding effects are known to influence protein behaviours^29^ and yet, remarkably, virion maturation consistently produces similar capsid morphologies. The relatively fast maturation process^30^,^31^ makes *in vivo* study of capsid self-assembly pathways difficult. Nonetheless, Woodward *et al*.^32^ show capsid formation proceeding via a hook-shaped precursor to the broad end of the capsid, in contrast with models of capsid assembly that begin with the narrow end of the cone^33^.

As the average virion contains approximately twice the CA present in a mature capsid, a capsid exists in equilibrium with a relatively high concentration of solution-state CA. Viral infection requires the transfer of virion contents into a target cell, producing a rapid dilution of this CA solution. Rapid dilution destabilizes the CA lattice *in vitro* under physiological salt concentrations^12^, but the potential significance of this effect on capsid stability is unclear.

Computer simulations of CA self-assembly offer a valuable complement to other experimental techniques. Atomic-resolution molecular dynamics (MD) simulations of pre-assembled capsids have been reported for HIV-1 (ref. 22), but the long-time stability of such MD models has not been demonstrated and their great computational expense prevents such models being used to examine capsid self-assembly. Coarse-grained (CG) models using simpler molecular representations^34^,^35^ provide an appealing alternative; for example, extensive CG simulations of icosahedral capsid self-assembly have been reported^36^,^37^,^38^,^39^,^40^, but the small, well-defined end products are in striking contrast to the structural pleomorphism observed with HIV-1 CA. Non-equilibrium CG simulations featuring irreversible subunit binding can generate capsid-relevant morphologies^41^,^42^, but may struggle to represent any dynamic reorganization that might occur at the leading edges of lattice growth. The CG self-assembly of CA dimers was explored using Monte Carlo^43^ and MD simulations^34^ on two-dimensional or quasi-two-dimensional surfaces, and recent work extended Monte Carlo simulations to three-dimensional (3D) systems^44^ albeit using rigid models that cannot capture the innate NTD/CTD flexibility of the capsid protein.

In this work, we consider the nucleation and growth of CG HIV-1 capsid protein in 3D systems under various conditions relevant to the viral lifecycle. In particular, we examine the effects of CA concentration and molecular crowding on mature CA lattice generation, the influence of dynamic transitions between populations of CA that are compatible and incompatible with mature lattice formation, and the effects of rapid CA dilution on previously self-assembled CA lattice structures. While comparisons to fully assembled HIV-1 capsid structures from experimental data are difficult (our system sizes are restricted by computational expense), and hence caution is warranted when considering large-scale structural assemblies, the early stages of nucleation and growth for capsid protein lattice are accessible in our simulations. In total, we present the results of 51 separate CG simulations and examine general trends across the data sets.

## Results

### The effects of CA concentration and molecular crowding

As noted, there is a dynamic population of CA conformations dictated by the flexible linker between the NTD and CTD. For our simulations, the equilibrium NTD-CTD conformation was based on the curved hexagonal lattice within CA tubes (Supplementary Fig. 1). Coarse-grained molecular dynamics (CGMD) simulations were performed (see Methods and Supplementary Fig. 1A–C) using CA concentrations [CA] of 1, 2, 3 and 4 mM (with [CA] in a typical virion estimated as ≈3.8 mM)^24^. For each [CA] studied, inert CG crowding agent densities *ρ*_CR_ from 0 to 200 mg ml^−1^ were present with increments of 50 mg ml^−1^ to study the effects of molecular crowding on CG self-assembly^12^. A single simulation was performed for each data point for 2 × 10^8^ MD time steps, with results summarized in Table 1 (full data in Supplementary Fig. 2).

For [CA]=1 mM, no stable self-assembly of mature-style capsid lattice occurred for any level of crowding studied. Instead, populations of metastable ‘trimer-of-dimers' structures^34^ rapidly formed (Fig. 2a,b), with average trimer populations increasing with *ρ*_CR_. A similar trend was observed for [CA]=2 mM until 200 mg ml^−1^ of crowder was present, at which point the self-assembly behaviour changed dramatically. Rather than generating a steady-state population of trimers, as was apparently the case for *ρ*_CR_=150 mg ml^−1^, a new self-assembly regime emerged: the steady production of mature-style CA lattice with quasi-equivalent pentamers and hexamers. Similar effects were observed for [CA] of 3 and 4 mM, with metastable trimers generated below the onset of CA lattice growth at 100<*ρ*_CR_≤150 mg ml^−1^ (for [CA]=3 mM) and 50<*ρ*_CR_≤100 mg ml^−1^ ([CA]=4 mM). The transition from producing only trimers to the nucleation of mature-style lattice was thus a function of both [CA] and *ρ*_CR_, with higher [CA] requiring lower *ρ*_CR_ to induce lattice growth. Differences of ≤50 mg ml^−1^ in *ρ*_CR_ therefore led to markedly different behaviours for [CA]≥2 mM.

Where simulations generated only steady-state trimer populations, the steady state was rapidly attained (<5 × 10^6^ MD time steps). For simulations producing mature-style CA lattice, the appearance of stable pentameric inclusions lags the production of stable hexamers (see Supplementary Fig. 2). In every simulation producing mature-style lattice, nucleation and growth of multiple lattice regions occurred. For example, with [CA]=2 mM and *ρ*_*CR*_=200 mg ml^−1^, two independent lattice regions were generated: an incomplete cone fragment, and a small array of hexamers (Fig. 2c). For [CA]=3 mM and *ρ*_CR_=200 mg ml^−1^, several independent lattice regions appeared, resulting in a pill-shaped structure with a closed lattice surface and the fusion of an incomplete pill shape with a cylinder fragment (Fig. 2d). For [CA]=4 mM and *ρ*_CR_=200 mg ml^−1^, multiple lattice regions fused into a semi-amorphous assembly (Fig. 2e), reminiscent of multiple capsid structure aggregations observed in electron cryotomograms (Fig. 2f,g)^45^. The CG model is thus capable of producing a wide variety of curvatures, as required by the continuous curvatures observed in pleomorphic HIV-1 capsids.

Given CA molecular mass≈25 kDa, [CA] of 2, 3, and 4 mM correspond to CA mass densities of ≈50, ≈75 and ≈100 mg ml^−1^. Onset of mature-style lattice growth therefore occurred for total mass densities of between 200 and 250 mg ml^−1^ (for [CA]=2 mM), 175 and 225 mg ml^−1^ ([CA]=3 mM), and 150 and 200 mg ml^−1^ ([CA]=4 mM). Total non-lipid mass density of HIV-1 virions was estimated as ≈200–300 mg ml^−1^ (ref. 24), and so the CG model self-assembly initiated at mass density levels comparable to the virion.

These results indicate sensitivity to both [CA] and local molecular crowding for CG self-assembly. Well-regulated nucleation and growth pathways are therefore critical for capsid production, given significant natural variability in HIV-1 virions^24^,^25^,^26^,^27^,^28^.

### Effects of CA concentration with constant molecular crowding

Inert molecular-crowding effects can significantly alter CG self-assembly. However, CA is also a self-crowding agent as it occupies volume in the simulations. Direct comparison between self-assembly behaviours of different [CA] with the same quantity of inert crowder is therefore non-trivial.

To examine the effects of CA concentration on CG self-assembly under constant molecular crowding, the initial CA content of identical systems (4 mM CA, *ρ*_CR_=200 mg ml^−1^) is partitioned into fixed ‘active' ([CA]_+_) and ‘inactive' ([CA]_−_) populations. After the initial partitioning, CA dimers remain either ‘active' or ‘inactive' for the duration of the simulation. This partitioning is motivated by the experimental results of Deshmukh *et al*.^23^ suggesting a relatively small population of ‘assembly-competent' CA dimers in solution. Inactive CA is identical to active CA in all aspects in our CG model except for the removal of attractive interactions, and thus both types of CA occlude equivalent volume. By fixing [CA]_+_ between 250 μM and 4 mM (with increments of 250 μM), CG self-assembly is studied as a function of CA concentration alone while maintaining fixed, virion-relevant crowding levels. The formation of trimer-of-dimers, hexamer, and pentamer structures are recorded over 2 × 10^8^ CGMD time steps (one simulation per CA concentration), with selected results summarized in Fig. 3 (full data in Table 2 and Supplementary Fig. 3).

For 250≤[CA]_+_≤500 μM, no generation of mature-style CA lattice occurs, but metastable trimer-of-dimer populations emerge. Elevating [CA]_+_ to 750 μM produced an apparent initial steady-state trimer population before a single region of mature-style lattice nucleated and grew after ≈4 × 10^7^ MD time steps. With [CA]_+_=1 mM, the nucleation and growth of a single-lattice region began after ≈1 × 10^7^ MD time steps. For both [CA]_+_=750 μM and [CA]_+_=1 mM, the lattice region adopted a cylindrical conformation as growth proceeded; although pentameric inclusions regularly formed at the growing edge of the lattice, the inclusions were transient and thus the lattice was essentially hexameric. For all [CA]_+_≥1.25 mM nucleation and growth of multiple lattice structures occurred, with regions of high local curvature around the pentamers. Also evident at higher [CA]_+_ were lamellar regions of CG lattice (Fig. 3) reminiscent of structures observed in electron cryotomograms. Lamellar and off-pathway capsid structures are indicated to be significantly more common in virion systems lacking RNA/NC complex^46^, as is the case in these CG simulations.

The relatively controlled nucleation and growth for lower [CA]_+_ reveals specific details of CG lattice nucleation. Visual inspection of simulation trajectories indicates lattice nucleation occurs by the addition of CA dimers onto existing trimer-of-dimers structures, producing trimers with shared edges (Fig. 4) and leading to a central hexamer stabilized by a peripheral trimer ‘skirt' from which lattice growth proceeds. Metastable trimers therefore appear to seed the nucleation and growth of CG lattice^34^, rather than independent trimers aggregating. Transient structures along this pathway are observed in simulations that do not produce mature lattice, but these intermediates are unstable and dissociate before nucleation of lattice growth.

Examination of the solution-state [CA]_+_ reveals that lattice growth continues below the level of solution-state [CA]_+_ required for initial nucleation (for example, Fig. 5a), in agreement with standard nucleation and growth theories^47^. As a region of mature lattice grows, there is also a reduction in the number of separate trimers in solution (for example, Fig. 5b). Progressive reduction of not only the ‘available' capsid protein but also the number of trimers in solution (the potential nucleating seeds) can thus provide an elegant feedback mechanism to deter multiple capsid formation.

These results indicate a marked sensitivity of CG self-assembly processes to CA concentration under constant molecular crowding. Increases of as little as 250 μM in [CA]_+_ can drive the system from steady-state populations of metastable trimer structures ([CA]_+_<750 μM) into the nucleation and growth of single (750 μM*≤*[CA]_+_≤1 mM) or multiple lattice regions ([CA]_+_≥1.25 mM) over the timescales examined. Greater quantities of [CA]_+_ may encourage pentamer formation and/or stability over the course of the simulations: for [CA]_+_≤1 mM, no stable pentamers were detected (Table 2).

### The effects of NTD/CTD conformational freedom

The NTD and CTD structures of CA are well conserved, but a flexible linker region allows significant conformational freedom in solution. Deshmukh *et al*.^23^ estimate that the solution-state CA with NTD/CTD arrangements compatible with mature lattice might be as low as ≈5%. Furthermore, this population is inherently dynamic, with correlation times for inter-domain motions estimated as between 2 and 5 ns (ref. 23). The CGMD simulations with fixed populations of ‘active' ([CA]_+_) and ‘inactive' ([CA]_−_) CA effectively describe a limiting case of this behaviour: [CA]_+_ corresponds to a population of CA whose instantaneous structure is compatible with mature lattice (and hence may directly self-assemble) and the fixed populations of [CA]_+_ and [CA]_−_ correspond to infinitely long correlation times for inter-domain motions (that is, specific proteins remain in either assembly-competent or -incompetent conformations for the entire simulation).

The effects of interconversion between structural populations are now considered. Rather than initial fixing of [CA]_+_ and [CA]_−_ populations, all ‘free' CA in solution (that is, CA which is not currently aggregated) is periodically identified and then randomly assigned to [CA]_+_ or [CA]_−_ populations to maintain a specific target ratio of [CA]_+_ and [CA]_−_ in solution. The interval between random population assignments is thus a simplified proxy for time correlations in CA structural conformation: once assigned to either the [CA]_+_ or [CA]_−_ populations, a CA dimer remains ‘assembly-competent' or ‘assembly-incompetent' at least until the next random population assignment. Aggregated CA is not included in this process to avoid perturbation of existing aggregates, and to reflect CA conformational stability in mature CA lattice. Specific CA can disaggregate and, in the absence of re-aggregation, is subject to the next random population switch. This approach allows significant control over the quantity of assembly-competent CA in solution to mimic the coexistence of assembly-competent and -incompetent populations.

For an identical baseline system (4 mM total CA, 200 mg ml^−1^ crowder), target ‘assembly-competent' proportions [CA]_%_ of 2.5, 5, 10, 25 and 50% of solution-state CA were studied (one simulation per [CA]_%_ value). Random population switching was performed with intervals of 1 × 10^4^, 1 × 10^5^ and 5 × 10^5^ CGMD time steps, corresponding to 0.1, 1 and 5 ns of CG time respectively, but caution should be exercised in any direct comparison of CG timescales to those suggested by NMR experiments (see note regarding CG timescales in Supplementary Note 4). Simulations were performed for 2 × 10^8^ MD time steps unless explicitly noted, with results summarized in Table 3 and Fig. 6 (full data in Supplementary Fig. 4).

Apparent from Table 3 is the influence of conformational switching intervals on CG self-assembly. For the most rapid switching rate (interval=1 × 10^4^ time steps), no self-assembly of mature-style CA lattice emerges until some 50% of the total CA in solution is considered to be assembly-competent: below this level, only metastable trimer-of-dimer structures appear. With a slower switching rate (interval=1 × 10^5^ time steps), the transition from steady-state trimers into mature-style lattice occurs for 10%<[CA]_%_≤25%. For the slowest switching rate studied (5 × 10^5^ time steps), the transition occurs at 5%<[CA]_%_≤10%. Where simulations produced only metastable trimers, faster switching rates reduced the average trimer population for identical [CA]_%_ (with an additional 1 × 10^8^ MD time steps performed to improve statistical estimates). These results suggest that NTD/CTD correlation times affect CG self-assembly, with faster switching between assembly-competent and -incompetent forms suppressing lattice formation for otherwise identical [CA]_%_.

When a target active proportion [CA]_%_ of 10% was combined with a switching interval of 5 × 10^5^ time steps, a cone-shaped structure reminiscent of a mature capsid was produced (Fig. 6). A small region of hexameric lattice initially formed, which curled into a semi-cylinder under growth. Stable pentamer incorporation appeared alongside higher local curvatures at one end of the structure, generating the narrow end of a cone. At this point lattice growth essentially stopped because of insufficient assembly-competent CA in solution, as the system contained only 50% of the CA content of a typical virion^24^,^25^,^26^. Although transient pentameric inclusions regularly formed at the exposed lattice edges, all permanent capsomer additions were hexameric after this point. It is interesting to note that pentamers were occasionally embedded slightly behind the growing edge of the lattice, but were replaced by hexamers in a process of local remodelling. The CG self-assembly can thus demonstrate a degree of ‘error correction', provided the growing lattice edge does not advance too far beyond the pentamers. A repeat simulation was performed using a different initial momentum distribution with quite similar results (Supplementary Fig. 5), but it should not be inferred that these specific conditions are unique in producing this outcome.

### Stability and uncoating of self-assembled CG structures

Capsid assembly is critical for HIV-1 infectivity, but capsid destruction is also crucial to the viral lifecycle. Successful infection is temporally sensitive to capsid uncoating after cell entry, with both premature and delayed uncoating detrimental to viral replication^6^,^48^. Assuming a virion radius ∼630 Å (ref. 24), the transfer of virion contents into a cell of radius≈10 μm (ref. 49) produces rapid dilution of the CA solution surrounding the capsid. To examine the effects of rapid dilution, simulations using previously self-assembled lattice structures were performed with [CA]_%_=0 to approximate the negligible background CA concentration after transferring virion contents into a cell. Importantly, this approach preserves the level of molecular crowding and conformational switching rate under which the lattice structures originally formed, allowing investigation of rapid dilution on several different structures that are otherwise stable under identical conditions. This avoids the use of a single model structure, whose natural stability may differ subtly under different test conditions even in the absence of rapid dilution. The simulations therefore probe the effects of rapid dilution specifically, representing a cytoplasmic environment that is otherwise identical to the notional virion in which the structures were generated.

**Figure 1:**
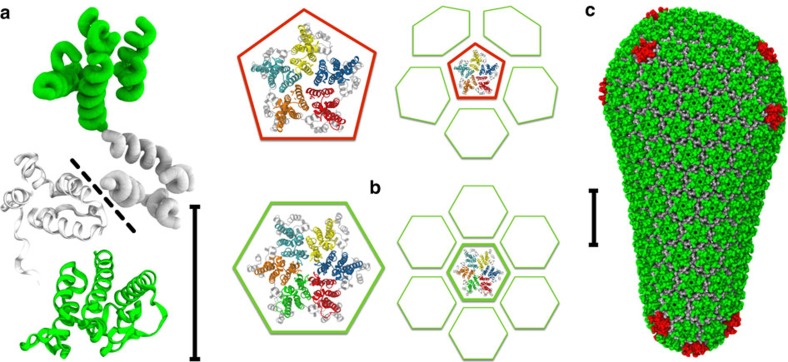
The fullerene cone model of the HIV-1 capsid is assembled from ‘mature-style' capsid lattice with hexameric (green) and pentameric (red) building blocks. (**a**) CA dimer structure with both CG (tube) and all-atom (ribbon) monomer representations (NTDs green and CTDs grey). CTD dimer interface marked by dashed line; scale bar, 5 nm. (**b**) Quasi-equivalent pentamer and hexamer capsid inclusions (NTDs distinguished by colour, all CTDs grey), with schematic of adjacent packing in capsid lattice. (**c**) Mature capsid structure after Pornillos *et al*.^21^. NTDs of hexamer-associated CA shown in green, with NTDs of pentamer-associated CA shown in red (all CTDs grey); scale bar, 20 nm.

**Figure 2:**
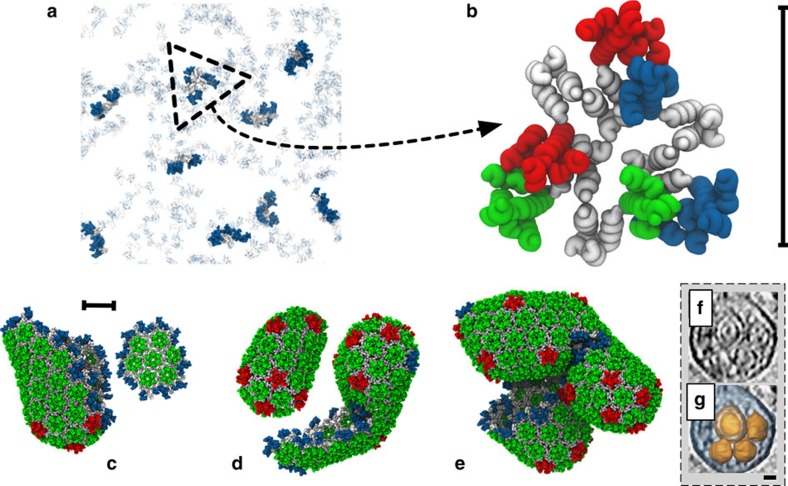
Dimers of the CA protein are the units of self-assembly of HIV-1 capsid-like CG structures. (**a**) Simulation snapshot of a population of metastable trimer-of-dimers. CA NTDs are blue, with CTDs grey (non-aggregated CA shown transparent for clarity). (**b**) Detail of a CG trimer-of-dimers, where ‘edges' are CA dimers, with two NTDs per triangle ‘vertex'. NTDs coloured by dimer for clarity, scale bar 10 nm. Final simulation snapshots for *ρ*_*CR*_=200 mg ml^−1^ and [CA]=2 mM (**c**), 3 mM (**d**) and 4 mM (**e**) are presented with NTDs coloured by monomer presence in a trimer (blue), pentamer (red) or hexamer (green). All CTDs grey, scale bar 20 nm. (**f**) Multiple aggregated capsid structures as revealed by electron cryotomography, with capsid structures highlighted in orange (**g**). Scale bar, 20 nm; **f** and **g** adapted from ref. 45.

**Figure 3:**
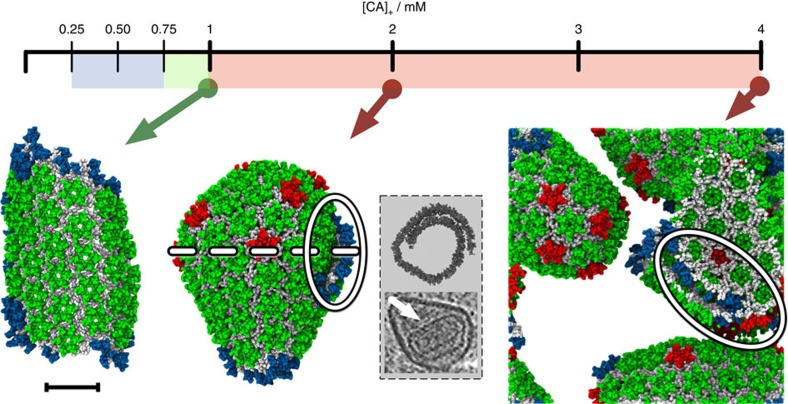
Only a narrow range (indicated in green) of CA concentrations results in the nucleation and growth of a single-lattice region. CG self-assembly as a function of active CA concentration [CA]_+_ under fixed crowding conditions. Colour in the concentration bar indicates formation of only trimer-of-dimers structures (blue) and the nucleation and growth of single (green) or multiple (red) lattice regions. Example final structures are shown for [CA]_+_=1, 2 and 4 mM (arrows). CA colour scheme as in Fig. 2, lamellar regions highlighted by ovals. Final structure for [CA]_+_=2 mM formed via two lattice regions fusing. Grey panel shows cross-sectional slices (not to scale) to illustrate lamellar lattice in structure for [CA]_*+*_=2 mM (top, cross-sectional plane indicated by dashed white line) and an example lamellar CA lattice inside a virion from electron cryotomography (bottom, CA lattice indicated by white arrow). Final structures for [CA]_+_=4 mM wrap around periodic boundaries. Scale bar, 20 nm.

Maximally assembled systems (that is, using [CA]_%_=50%) for the three conformational switching rates described previously were used as starting configurations for rapid dilution, with [CA]_%_ now set to 0% in each case. Figure 7 presents the resultant time series of structural data from one rapid dilution simulation per starting conformation, with CA lattice dissolution evident in all systems. Conformational switching intervals of 1 × 10^4^ time steps (Fig. 7a) and 1 × 10^5^ time steps (Fig. 7b) show a distinct and rapid destruction of any remaining lattice at ≈175 × 10^6^ and ≈550 × 10^6^ CGMD time steps, respectively, indicating lattice dissolution is not a simple function of time (lattice structures at these points shown in Fig. 7, inset). Interestingly, the 5 × 10^5^ time step switching interval showed a long-lived plateau between ≈350 × 10^6^ and ≈800 × 10^6^ MD time steps corresponding to reorganization of the remaining lattice into a pill-like structure with closed surface (Fig. 7c). This enhanced stability is because of the absence of exposed lattice edges: CA in edge-free lattice requires significant reorganization of surrounding lattice components to escape, but can dissolve from exposed edges directly.

These results demonstrate the instability of CG capsid lattice under rapid dilution, even under conditions otherwise identical to those in which the lattice assembled. Furthermore, the results suggest that CA lattice structures with no exposed edges may be significantly more stable upon transfer into a cell.

## Discussion

**Figure 4:**
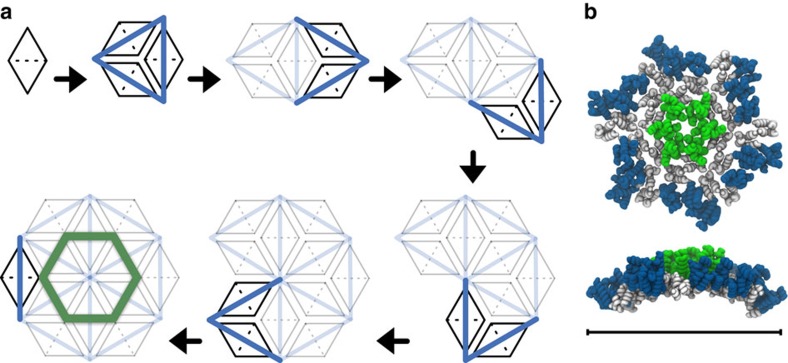
Putative assembly from 12 CA dimers that nucleates CG capsid lattice growth. (**a**) Reversible addition of CA dimers (CA monomers depicted as black triangles, CTD/CTD interface as a dashed line) onto existing aggregates produces trimers with shared edges, eventually generating a central hexamer stabilized by trimer ‘skirt'. (**b**) Front and side view of example structure from CG simulation, with mild innate curvature visible. Hexamer-associated NTDs are green, NTDs in trimer skirt blue, CTDs grey. Scale bar, 20 nm.

**Figure 5:**
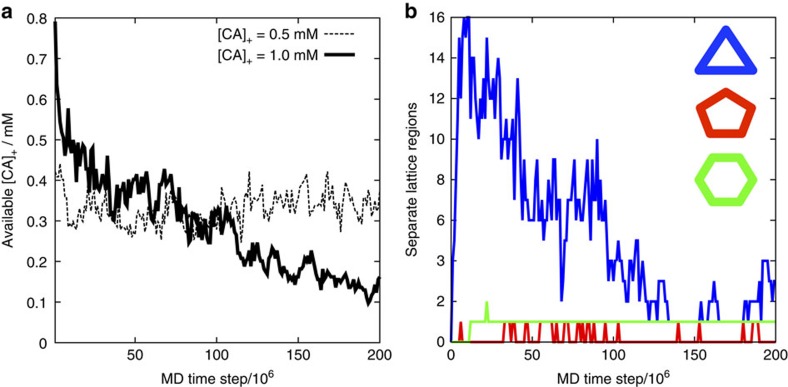
Example CG simulation data under fixed molecular crowding. (**a**) ‘Available' assembly-competent CA in solution for initial [CA]_+_=0.5 mM (resulting in no nucleation and growth of mature lattice) and initial [CA]_+_=1.0 mM (producing nucleation and growth of a single-lattice region, see the main text and Table 2). Lattice growth continues below the level of available [CA]_+_ required for nucleation on the same timescale. (**b**) Number of separate lattice regions containing key structural motifs for [CA]_+_=1 mM. The number of trimers in solution (blue curve) reduces significantly as a single region of lattice grows.

Our CG model is capable of self-assembly into structures that reproduce native protein/protein interfaces in the mature HIV-1 capsid lattice HIV-1 capsid lattice, including subtle effects such as quasi-equivalent NTD packing in pentameric and hexameric rings. While care should be taken in assuming any CG model to be a direct representation of a real, fully atomistically resolved system, the reproduction of specific experimental phenomena suggests that our CG model can capture the early and intermediate stages of nucleation and growth of the capsid lattice. Even so, we note that the computational expense still limits the system sizes considered in this work, preventing the generation of full capsid structures after the depletion of the CA concentration. Our study demonstrates that CG self-assembly is sensitive to CA concentration, molecular crowding, and NTD/CTD conformational correlation times. A single CG CA protein model generates surprisingly varied morphologies, encompassing the wide range of local curvatures required in the viral capsid^15^. Evident in the results is a reversible multi-stage process triggering mature lattice self-assembly^50^, where metastable trimer-of-dimers structures rapidly emerge to act as potential seeds for the nucleation of mature lattice growth (Fig. 4). The steady-state population of trimers increases as a function of both concentration and molecular crowding, until suitable conditions produce mature-style capsid lattice over the simulation lengths examined. Lattice growth progressively reduces both the solution-state CA and the population of independent trimers to deter the nucleation of additional lattice regions (for example, Fig. 5). The transition between self-assembly regimes appears to be quite stable, in which relatively small changes in the molecular environment are capable of producing different outcomes, in agreement with, for example, previous studies of self-assembly for icosahedral capsid models^37^,^38^,^39^,^40^,^51^,^52^.

The general effects of molecular crowding in our simulations are in agreement with those observed using other computational approaches for self-assembly. For example, high levels of crowding can reduce nucleation lag times to effectively zero (see, for example, Supplementary Figs 2–4), changing the self-assembly process from nucleation-limited to non-nucleation-limited. Smith *et al*.^39^ also noted such effects in discrete event simulations of icosahedral capsid self-assembly: multiple parallel nucleation and growth events depleted the available capsid components, arresting complete capsid assembly and producing off-pathway growth. Similar kinetic traps and multiple nucleation were also observed by, for example, Johnston and co-workers in Monte Carlo investigations of icosahedral capsid assembly^37^,^38^,^39^,^40^,^51^,^52^.

In the absence of RNA, CA is typically observed to assemble into tubes of variable diameter *in vitro*^10^,^12^,^13^,^15^,^16^,^17^,^18^, albeit with occasional cone-shaped structures^12^ and other non-tubular morphologies^13^,^17^. These experiments typically feature CA concentrations significantly below that expected in a virion, which likely assists in producing regular, purely hexameric cylinders via orderly nucleation and growth.

Sigmoidal ‘assembly curve' characteristics have been observed in experimentally for capsid assembly in, for example, HIV-1 (ref. 12) and HPV-11 (ref. 53). Our results follow this general prediction, with assembly lag periods that are very short or effectively zero with suitable CA concentration and crowding (see, for example, Supplementary Note 2). We note that simulations lacking mature-style lattice production do not necessarily indicate that CG lattice growth cannot occur under those conditions, but rather that nucleation and growth were not detected over the simulation timescales examined. As the main focus of this study is the nucleation and growth of capsid lattice, converged simulations (that is, those attaining some final equilibrium) do not offer significantly more information regarding these aspects. Nonetheless, examination of structural data as a function of time (for example, Supplementary Figs 2–4) indicates that many simulations are approaching the plateau of a sigmoidal growth curve, with significant additional lattice growth unlikely.

Trimer-of-dimers structures have been reported previously in computer simulations^34^,^43^, and have also been assumed as the fundamental building block in mathematical models of capsid cylinder growth^54^. Our results suggest that trimer structures are metastable in isolation, but addition of CA dimers onto these templates (Fig. 4) provides the basic nucleation pathway for capsid assembly rather than the aggregation of independent trimer-of-dimer structures.

The regular appearance of transient pentamers at the growing edge of CG lattice was observed, suggesting a natural aspect of lattice growth. If growth is slow, local remodelling can remove the pentamers provided the expanding lattice edge does not advance too far. For more rapid assembly (for example, higher crowding or CA concentration) the lattice advances too quickly for reliable remodelling, producing high local curvature via stably embedded pentamers. This process can explain why relatively low concentrations of CA typically produce cylindrical structures *in vitro*^10^,^12^,^15^,^16^,^17^,^18^,^19^,^20^, and why increased CA concentration reduces cylinder length^16^: early pentamer incorporation redirects lattice growth, suppressing the formation of longer cylinders. Inherent instability in lattice edges suggest that CG models allowing dissociation more accurately reflect capsid assembly^41^,^42^,^50^.

Switching between active and inactive CA populations in solution, akin to the conformational dynamism recorded *in vitro*^23^, has pronounced effects on CG lattice self-assembly. Faster random mixing of the populations suppresses CG lattice production (Table 3), with a low instantaneous proportion of assembly-competent CA limiting uncontrolled nucleation and growth. Longer intervals between population switching might allow CA that dissociates from a lattice edge to remain assembly-competent long enough to reassociate directly, and thus maintain the overall lattice structure, but more investigation is required to elucidate this mechanism. In any case, dynamic conformational switching can ensure that growth is not prevented by the exhaustion of an otherwise limited quantity of assembly-competent CA. This suggests that CG models incorporating this phenomenon may better represent a key aspect of HIV-1 capsid assembly.

It is interesting to consider HIV-1 virion maturation in light of these results. Although the prototypical mature virion contains a single capsid, multiple capsid structures are sometimes observed^9^,^31^,^32^,^33^,^55^, both with and without RNA/NC encapsulation^32^, suggesting a delicate balance in the virion. Multiple capsids appear correlated with larger virions^33^,^55^, consistent with sensitivity of CG assembly to local conditions: lattice growth reduces CA in solution, so larger virions (with larger numbers CA for the same overall CA concentration) could maintain CA levels above the critical nucleating value after an initial nucleation, increasing the likelihood of subsequent nucleation events. This is compatible with observations that multiple cores are often comparable in size^55^, suggestive of separate nucleation events in quick succession. The sensitivity of CG self-assembly to CA concentration and local molecular crowding, in combination with natural virion variability, may complicate analyses that assume multiple cores are unrelated to CA concentration^55^.

**Figure 6:**
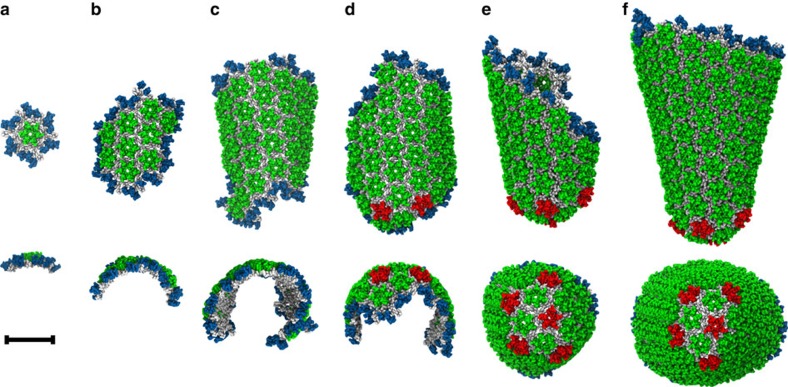
Steps in the assembly of the HIV-1 capsid by polymerization of CA dimers for [CA]_%_=10% and conformational switching interval of 5 × 10^5^ time steps (see the main text). Simulation snapshots at 120 × 10^6^ (**a**), 240 × 10^6^ (**b**), 440 × 10^6^ (**c**), 460 × 10^6^ (**d**), 600 × 10^6^ (**e**) and 1,700 × 10^6^ MD time steps (**f**) are shown with views perpendicular and parallel to the major structural axis. Colour scheme as in Fig. 2. Scale bar, 20 nm.

Our CG simulations produced virion-relevant morphologies such as the extended cone-shaped structure shown in Fig. 6. While the narrow end of this cone formed first, electron cryotomography indicates capsid growth typically proceeds from a small region of CA lattice adsorbed to the RNA/NC complex, generating the broad end of the capsid first (although capsid growth can also occur without RNA/NC)^32^. The presence of a RNA/NC complex would obstruct high local curvature (such as in the narrow end of a cone), selecting against the initial formation of a narrow end. Our simulations therefore do not fully recapitulate the virion environment, but instead explore principles relevant to capsid self-assembly. Nonetheless, the results are in general agreement with models of capsid generation where a small sheet of CA lattice curls under growth, producing a cup shaped structure from which assembly proceeds^32^.

Our results suggest the importance of relatively slow, controlled growth of the HIV-1 capsid, allowing significant relaxation of both the local and global structure to produce a metastable capsid despite weak interactions between CA dimers^11^,^12^. This phenomena suggests caution is appropriate when fitting atomic-resolution models into relatively low-resolution capsid data: for example, CTD/CTD dimer interfaces present in the atomistic MD simulations of HIV-1 viral capsids by Zhao *et al*.^49^ can display significant shearing and distortions from the expected structures^10^,^23^ (Supplementary Fig. 6). It is possible that such deformations occur naturally in a viral capsid, but further experimental support is needed to clarify this situation.

**Figure 7:**
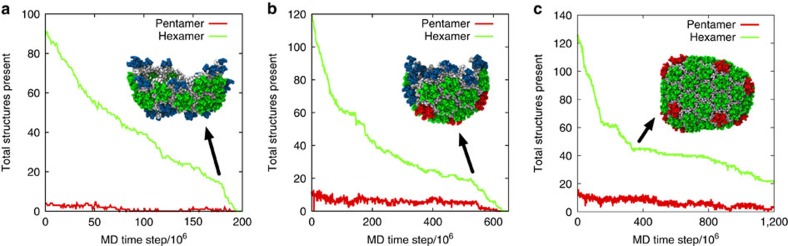
Disassembly of self-assembled CG CA lattice under simulated rapid dilution with constant molecular crowding. Conformational switching intervals of 1 × 10^4^ time steps (**a**), 1 × 10^5^ time steps (**b**) and 5 × 10^5^ time steps (**c**) are shown. Simulation snapshots (inset) correspond to the systems at ≈175 × 10^6^ (**a**), ≈550 × 10^6^ (**b**) and ≈350 × 10^6^ (**c**) CG MD time steps. Colour scheme as in Fig. 2.

The stability of CG lattice structures was sensitive to CA concentration in the supporting solution. Rapid dilution under otherwise identical conditions destabilized the capsid lattice, indicating the metastability of CA structures in agreement with experimental observations^12^. Similar effects have also been observed in simulations of icosahedral capsids^56^, albeit these simulations did not examine this effect under constant crowding conditions. Sealed, edge-free CA lattices appear more resistant to this process (Fig. 7c).

Cellular responses to HIV-1 include the weak binding of TRIM5α protein to capsids, forming a hexameric super-lattice on the capsid exterior^57^. TRIM5α binding promotes rapid capsid uncoating^48^,^58^,^59^,^60^,^61^,^62^,^63^, and while TRIM5α is digested by the proteasome^64^, capsid protein does not appear to be destroyed in this manner^58^,^59^,^63^. TRIM5α restriction is less efficient under proteasomal inhibition^65^,^66^,^67^, and we speculate this somewhat counterintuitive data can be explained by TRIM5α inducing local faults in the capsid lattice: slight mismatches in CA and TRIM5α lattice spacing induces additional stress in the metastable CA lattice^68^, with local tears providing an exposed edge from which CA can dissolve. Proteasomal degradation then ensures that a TRIM5α ‘scaffold' cannot exert any stabilizing influence on the overall capsid superstructure, enabling fragmentation of the capsid to further accelerate uncoating. Partial support for this hypothesis is offered by TRIM5α inducing only mild disruption of CA cylinders *in vitro*^61^, but as these studies used above-physiological salt concentrations with no rapid dilution (conditions in which the CA lattice may be over-stable)^12^ complete breakdown of CA lattice was not observed. Experimental data suggests HIV-1 capsids often feature exposed lattice edges, with an estimated 25% of cytoplasmic capsids featuring seams or holes large enough to allow the escape of green fluorescent protein (GFP)^42^, and so TRIM5α could help to accelerate natural lattice dissipation under rapid dilution. For capsid structures lacking exposed lattice edges, however, TRIM5α would provide a powerful accelerator of uncoating. This process would offer a relatively simple description of the general effects of TRIM restriction on HIV-a capsids, but we note that the natural TRIM-restriction processes could be significantly more complicated.

## Methods

### Model details

Full-model details provided in Supplementary Note 1. Briefly, CG CA is modelled directly from experimental hexamer (PDB 3H4E) and pentamer (3P0A) data^20^,^21^, and also heavily influenced by work from the same authors (B.K.G-P. and M.Y.) with cylindrical CA assemblies *in vitro*^14^,^15^,^16^,^19^,^20^. CA is represented as a dimer, the prevalent species in solution^10^,^23^, which is required for CA lattice assembly^11^. The NTD and CTD of a CA monomer are represented as independent stiff elastic network models (ENMs) comprising the C*α* atoms from CA-α helices, which are well conserved across experimental structures^34^. An additional weaker ENM connects the NTD and CTD in each monomer to provide limited inter-domain structural flexibility. The CTD/CTD dimer interface is represented by a stiff ENM based on PDB structure 2KOD (ref. 10) relevant for the mature lattice^23^. CG beads have excluded volume radii to deter unphysical overlaps as indicated by experimental structural data. Important CA protein/protein interfaces required for self-assembly^9^,^10^ are represented by specific attractive interactions, with the locations of energy minima parameterized using experimental structures^20^,^21^ and attractive strengths chosen to allow CA self-assembly under the experimentally relevant mass densities and conditions we study here (Supplementary Note 1). These attractive interactions can be dynamically enabled and disabled, allowing the generation of a fixed solution-state ratio of assembly-competent and assembly-incompetent populations as indicated by NMR data^23^. Inert crowding agent is based on the excluded volume and relative mass of Ficoll 70 (ref. 12). The combination of internal NTD/CTD flexibility in CA monomers, ‘switching' capability, and freedom of motion in a 3D simulation domain with explicit crowding molecules provides an advanced and relatively detailed molecular model with which to study CA self-assembly.

### Simulation details

All simulations were performed with our UCG-MD code^69^. The simulation domain was an 800 Å cube (≈50% of typical virion volume due to computational expense, with max. 616 CA dimers present at [CA]=4 mM) with periodic boundaries. Temperature of 300 K was maintained by Langevin dynamics with relaxation period 100 ps^−1^ to minimize influence of thermostat, integration time step 10 fs. Visualizations of simulation data were created using VMD^70^, with CG CA depicted as tubes connecting CG particles in each notional α helix to emphasize the local secondary structure of the CG model.

### Data availability

All relevant data are available from the authors on request.

## Additional information

**How to cite this article:** Grime, J. M. A. *et al*. Coarse-grained simulation reveals key features of HIV-1 capsid self-assembly. *Nat. Commun.* 7:11568 doi: 10.1038/ncomms11568 (2016).

## Supplementary Material

Supplementary Information Supplementary Figures 1-6, Supplementary Tables 1-4, Supplementary Notes 1-6 and Supplementary References

## Figures and Tables

**Table 1:**
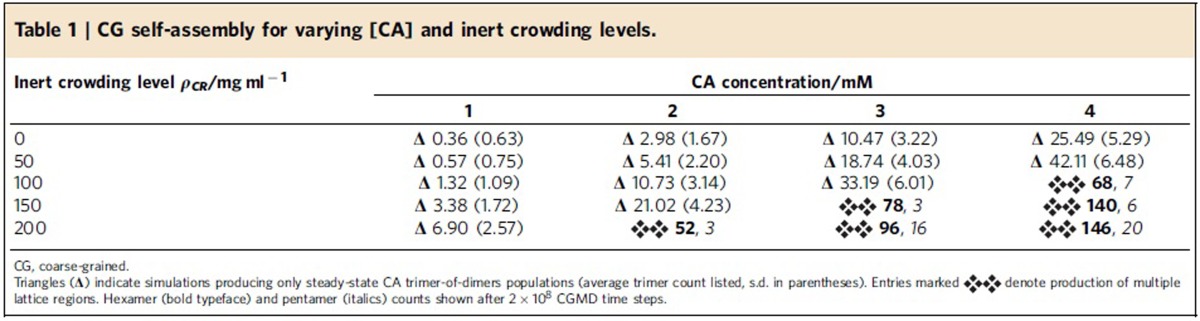
CG self-assembly for varying [CA] and inert crowding levels.

**Table 2:**
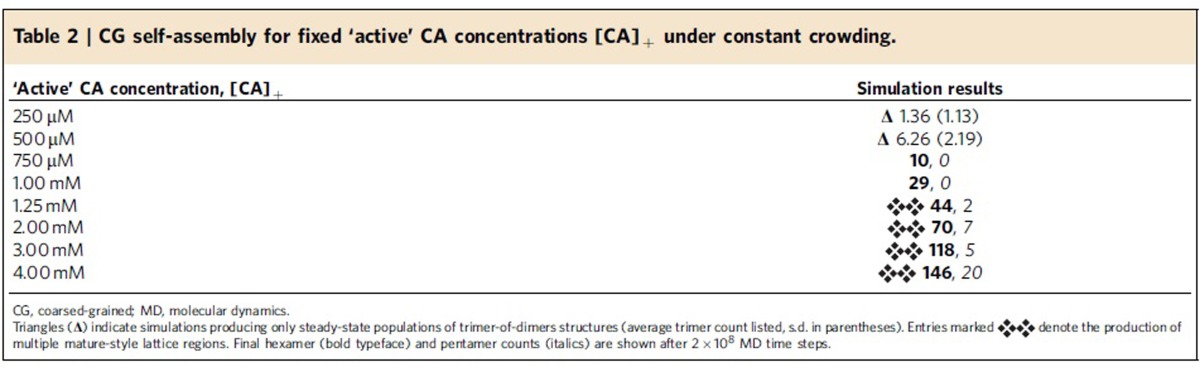
CG self-assembly for fixed ‘active' CA concentrations [CA]_+_ under constant crowding.

**Table 3:**
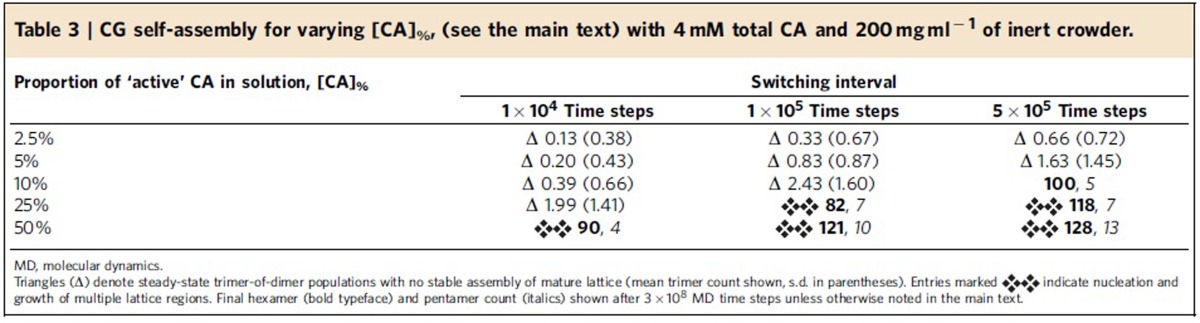
CG self-assembly for varying [CA]_%_, (see the main text) with 4 mM total CA and 200 mg ml^−1^ of inert crowder.
