# Supplementary material for: Coarse-grained simulation reveals key features of HIV-1 capsid self-assembly
Source: Nat Commun. 2016 May 13;7:11568. doi: 10.1038/ncomms11568 (PMC4869257; doi:10.1038/ncomms11568)
Supplement: Supplementary Information — Supplementary Figures 1-6, Supplementary Tables 1-4, Supplementary Notes 1-6 and Supplementary References [file ncomms11568-s1.pdf]

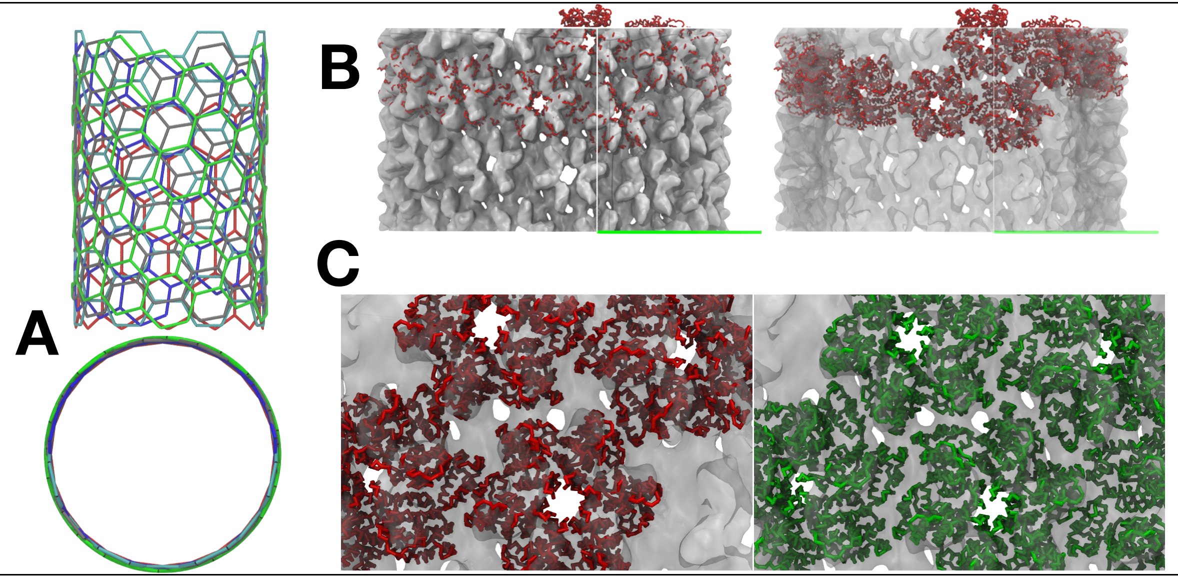

Supplementary Figure 1A. (A) superposition of example decoy geometries as seen from the side and top. (B) docking of a section of the minimum stress decoy structure into the EMD-5136 electron density map (shown with all-atom structures) before minimization with Langevin dynamics. (C) detail of a slice of the lowest stress decoy structure before (red) and after minimization (green) superposed onto the EMD-5136 electron density map (shown with all-atom structures).

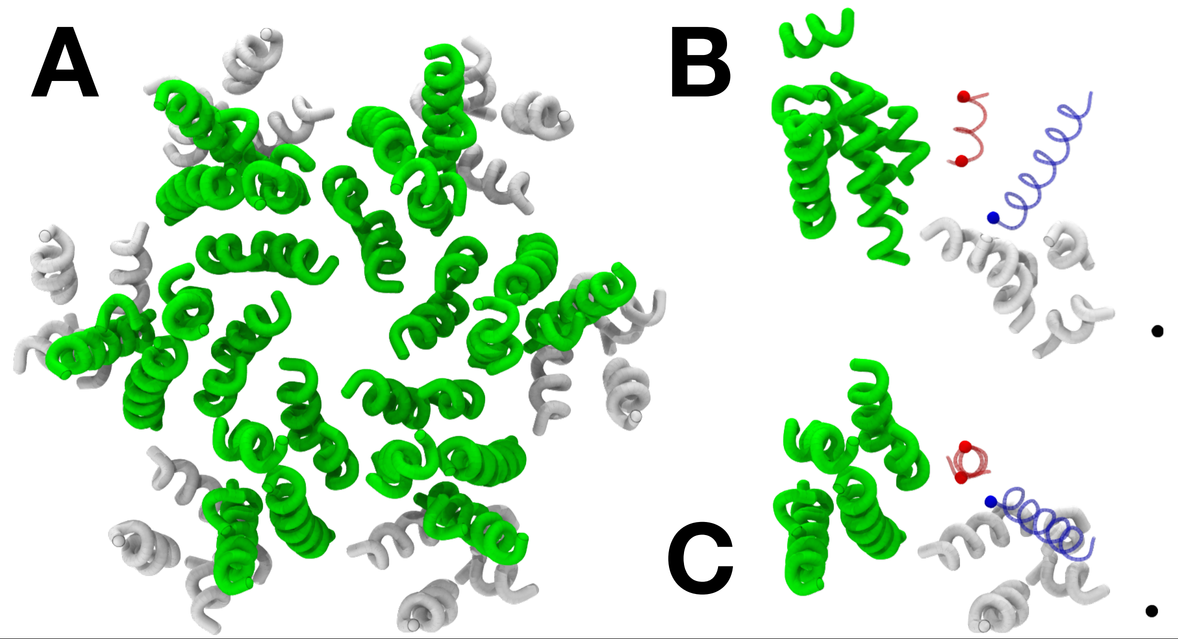

Supplementary Figure 1B. Structure of CG hexamer (from PDB 3H4E) and binding locations in the CG model. (A) CG model of PDB 3H4E, with NTDs green and CTDs gray. (B) Locations of helix 3 binding locations (red beads), helix 4 binding location (blue bead), and helix 10 binding location (black bead) shown relative to arbitrary CG monomer (thick tubes). Helix 3 of adjacent NTD in a hexameric conformation is shown as a red tube, helix 4 as a blue tube. (C) The same structures as (B), viewed from above.

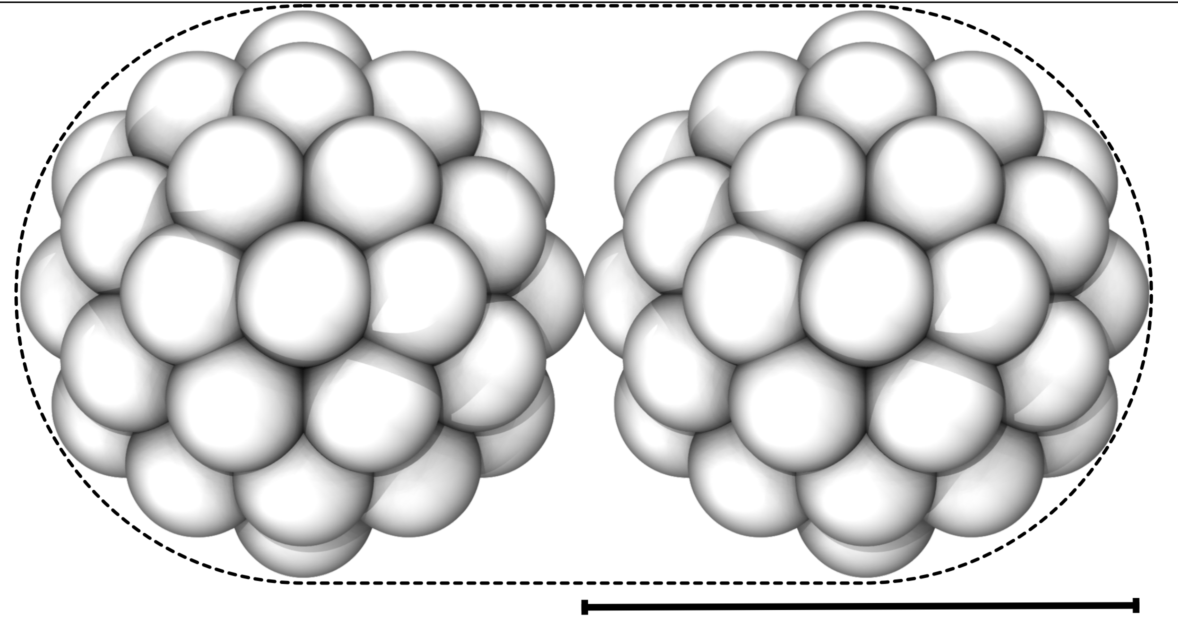

Supplementary Figure 1C. Inert CG crowding agent based on mass and excluded volume of Ficoll 70. Ficoll 70 geometry is intermediate to spherical and linear (e.g. dashed line), so the Stokes radius of Ficoll 70 (≈ 5.1 nm) is approximated by two spherical CG molecules of radius ≈ 2.5 nm. Scale bar 5 nm.

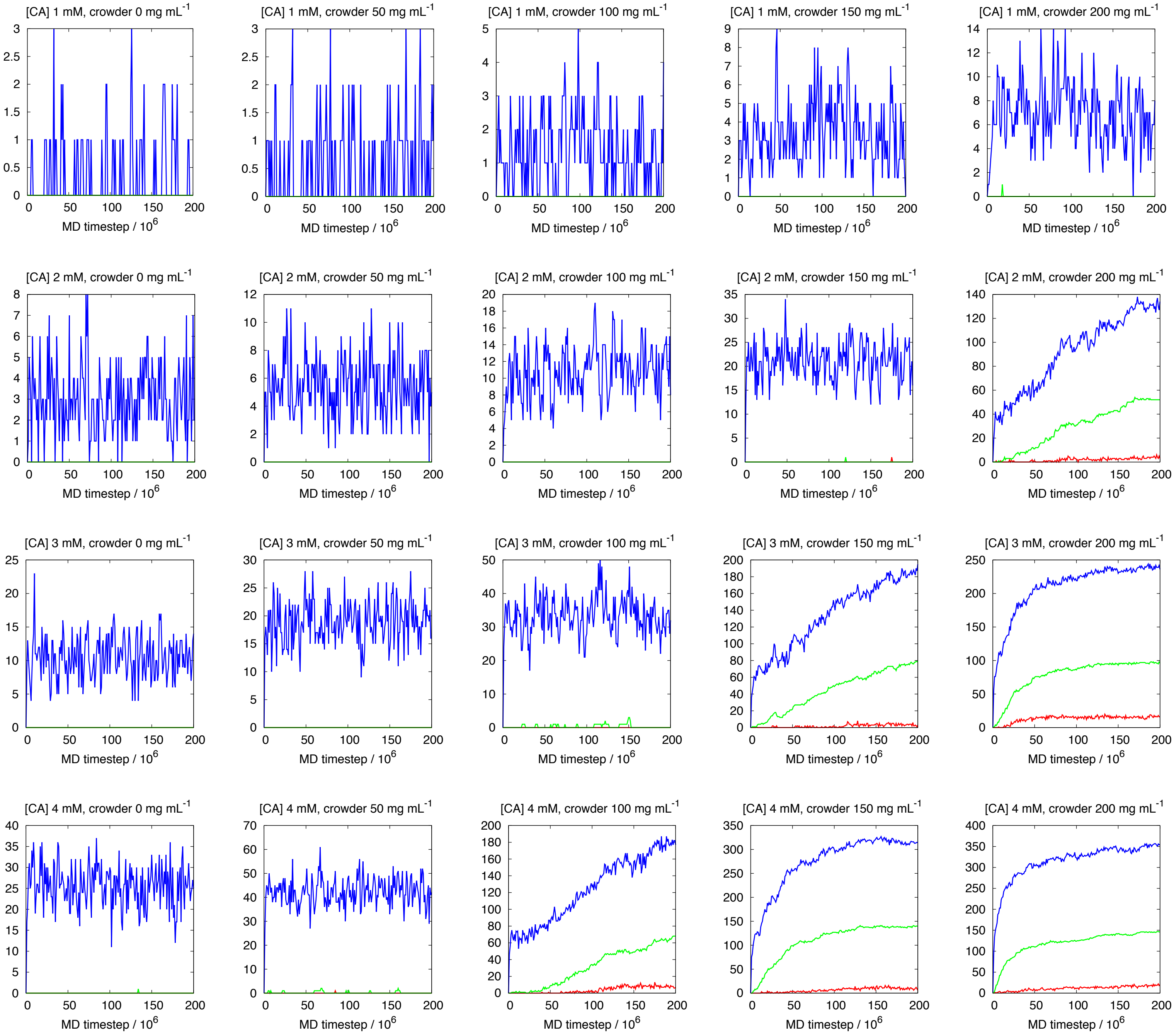

Supplementary Figure 2. Number of trimer-of-dimers (blue curves), pentamers (red) and hexamers (green) detected in CG MD simulations as a function of MD time step for various CA concentrations and quantities of inert crowding agent. Note that structure counts are not orthogonal: a trimer-of-dimers structure may also be a functional component of one or more hexamers or pentamers, and trimers may share “edges”.

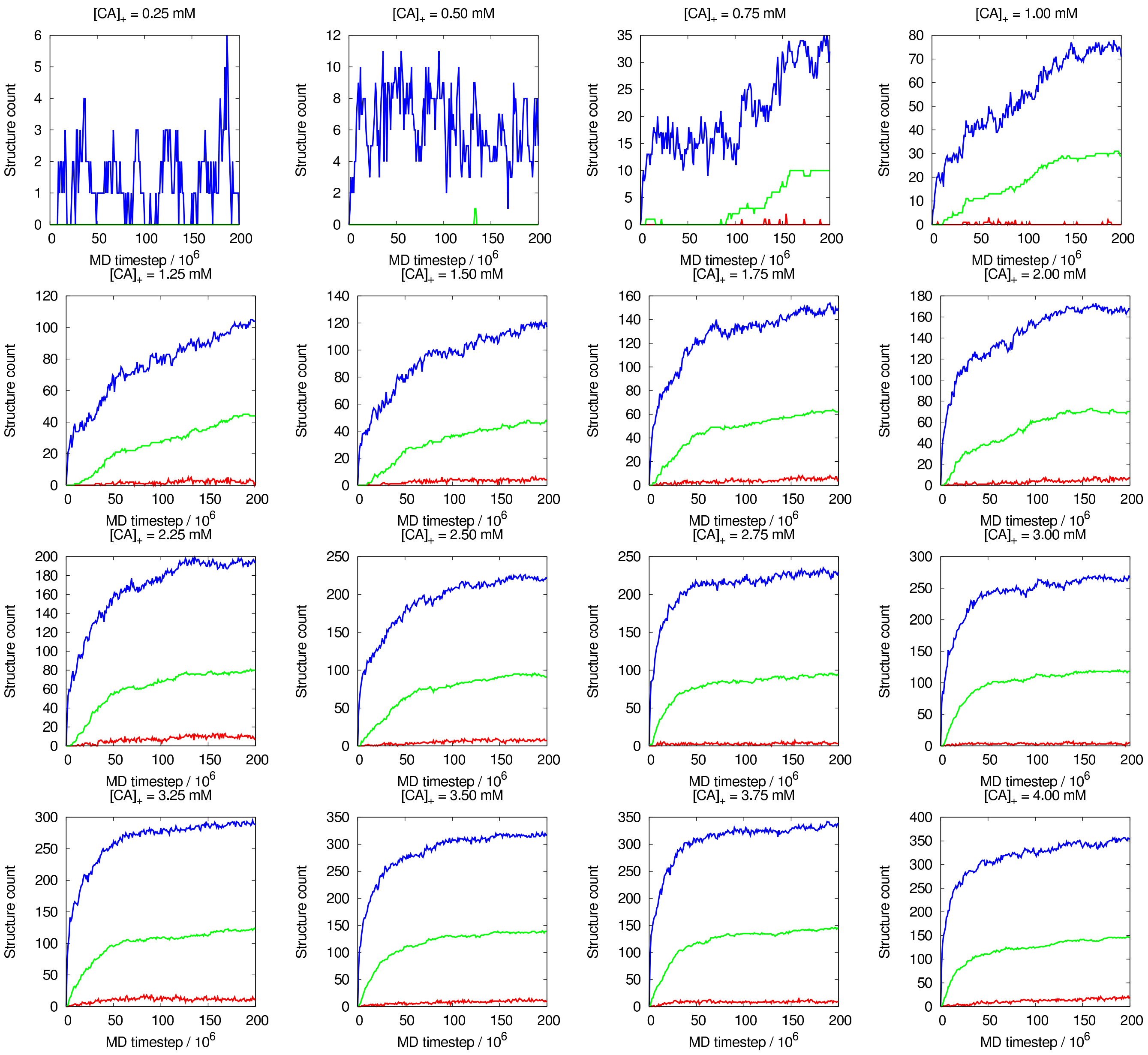

Supplementary Figure 3. Number of trimer-of-dimers (blue curves), pentamers (red curves) and hexamers (green curves) detected in CG MD simulations as a function of MD time step. Fixed crowding levels with fixed concentrations of “active” capsid protein (*$[CA]_+$*) were used (see main text). Note that structure counts are not orthogonal: a trimer-of-dimers structure may also be a functional component of one or more hexamers or pentamers, and trimers may share “edges”.

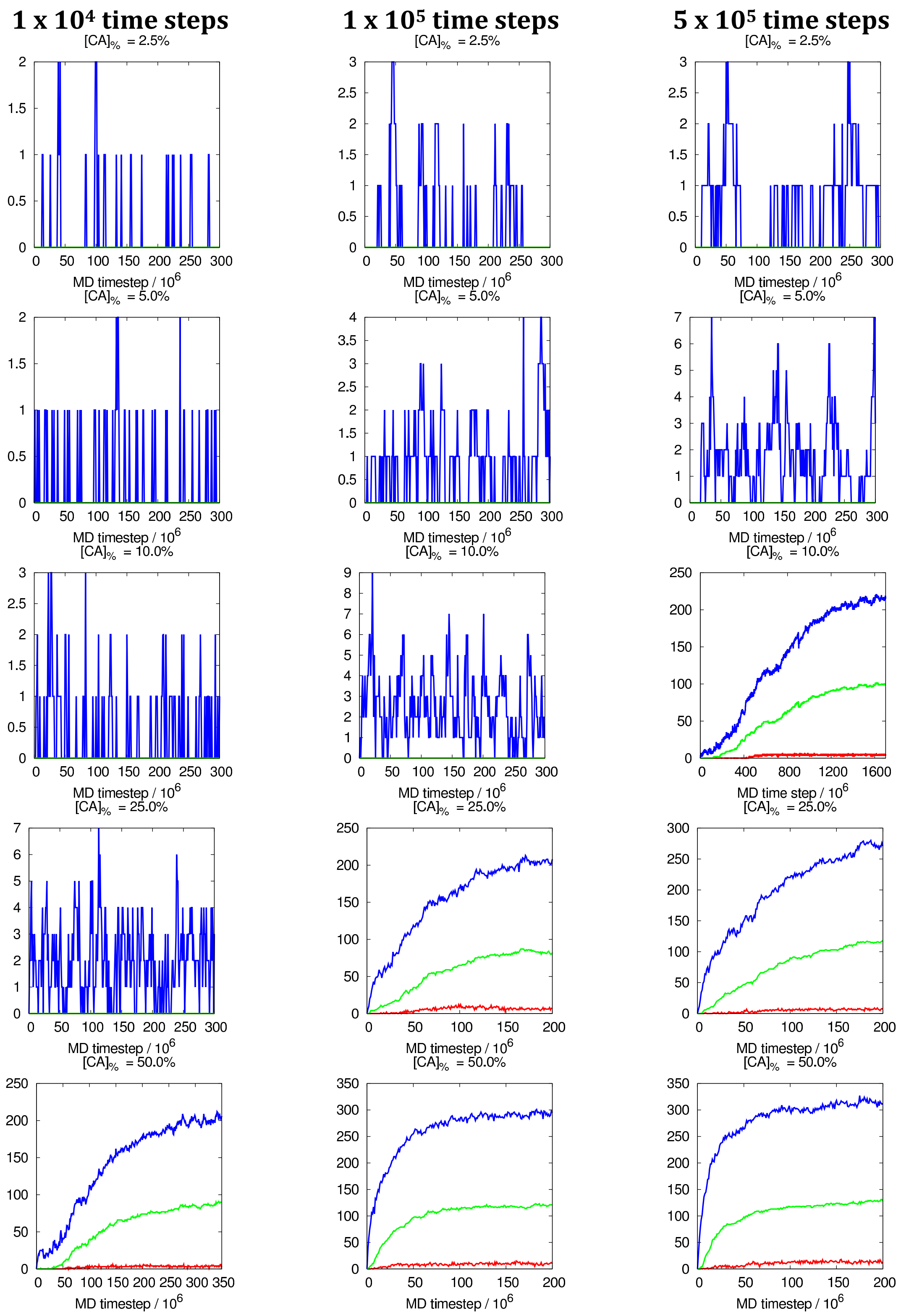

Supplementary Figure 4. Number of trimer-of-dimers (blue curves), pentamers (red) and hexamers (green) detected in CG MD simulations as a function of MD time step. Fixed crowding levels were used with the target proportion of "active" CA in solution ($[CA]_{\%}$) varied, along with the rate of conformational switching (see

main text). Note that structure counts are not orthogonal: a trimer-of-dimers structure may also be a functional component of one or more hexamers or pentamers, and trimers may share “edges”.

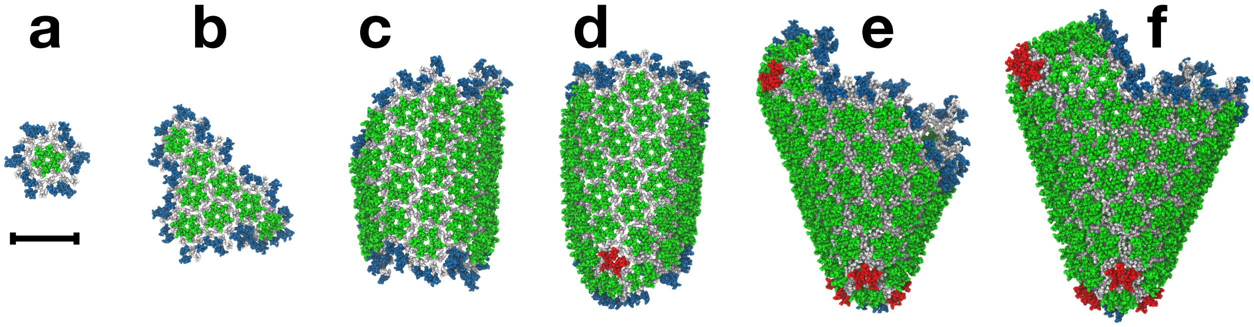

Supplementary Figure 5. Steps in the assembly of the HIV-1 capsid by polymerization of CA dimers for $[CA]_{\%}$ = 10% and conformational switching interval of 5 x 10^5 time steps (see main manuscript text and Fig. 6). This simulation was performed using the same initial configuration as used in Fig. 6 of the main manuscript, but a different starting momentum distribution was assigned to the CG particles. Simulation snapshots at 120 x 10^6 (**a**), 240 x 10^6 (**b**), 440 x 10^6 (**c**), 460 x 10^6 (**d**), 600 x 10^6 (**e**) and 1700 x 10^6 MD time steps (**f**) are shown. Colour scheme as in Fig. 6. Scale bar is 20 nm**.**

MD trajectory time (ns)	Example deviations from expected CTD/CTD dimer interface structure		
50	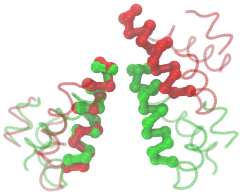	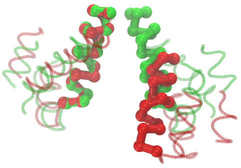	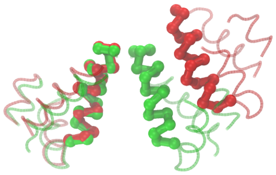
60	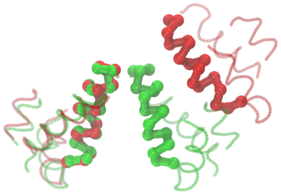	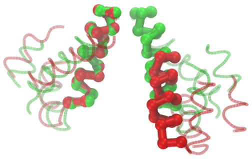	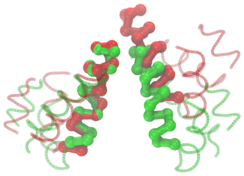
70	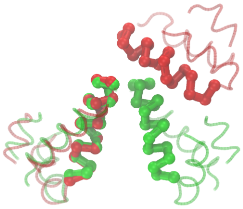	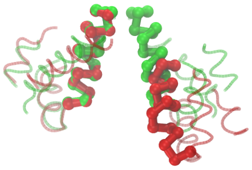	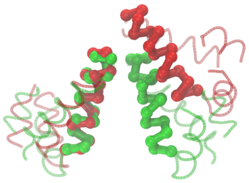
80	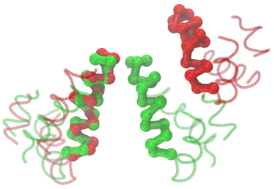	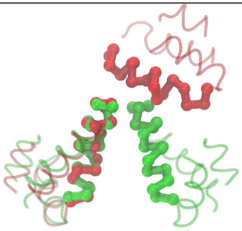	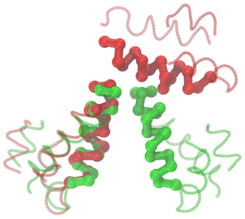
90	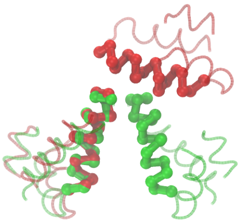	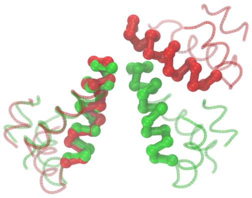	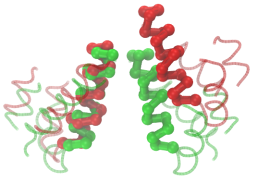
100	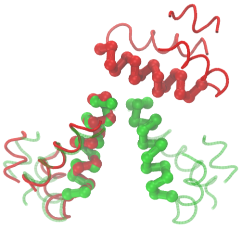	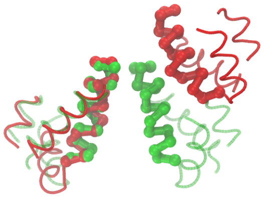	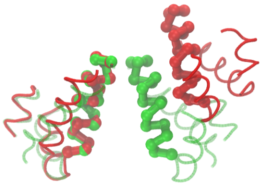

Supplementary Figure 6. Example CTD/CTD dimer interface configurations (red) from atomistic MD simulation [14] that differ significantly from the PDB structure 2KOD (green) [8]. Three example CTD/CTD dimer interfaces are shown for each trajectory snapshot, with α-helix 9 (wide ribbons) of one monomer superposed onto that of the 2KOD structure. Helices 8, 10, and 11 of each CTD are shown as thin transparent tubes, with all NTDs and CTD loop regions omitted for clarity.

Interface	Centroid residue(s)	3H4E RMSD	3P0A RMSD	Centroid separation
NTD/NTD	51<br>57	0.3 Å<br>0.2 Å	0.7 Å<br>0.5 Å	2.7 Å<br>1.3 Å
NTD/CTD	63	0.4 Å	0.6 Å	2.2 Å

Supplementary Table 1. CG particles selected for use in representing the NTD/NTD and NTD/CTD interfaces for self-assembly. The RMSD of centroid source data is listed separately for hexameric (3H4E) and pentameric (3P0A) experimental data. Also listed is the Cartesian separation between centroid locations calculated from the hexameric and pentameric data sets.

[CA], mM	Crowding, mg mL^{-1}	Cluster #1	Cluster #2
2	200	Sheet	Trough
3	150	Trough	Sheet
3	200	Sealed	Sealed
4	100	Sealed	Sheet
4	150	Sealed	Sealed
4	200	Sealed (multiple, fused)	(Nothing)

Supplementary Table 2. Morphological statistics from the largest ("Cluster #1") and second-largest ("Cluster #2") lattice regions identified at the end of the simulations shown in Supplementary Fig. 2.

$[CA]_+$, mM	Cluster #1	Cluster #2
0.75	Sheet	(Nothing)
1.00	Sheet	(Nothing)
1.25	Trough	(Nothing)
1.50	Trough	Sheet
1.75	Trough	Sheet
2.00	Sealed	(Nothing)
2.25	Trough	Trough
2.50	Sealed + trough	Sheet
2.75	Sealed + trough	(Nothing)
3.00	Sealed (multiple, fused)	(Nothing)
3.25	Sealed	Sealed
3.50	Sealed	Trough
3.75	Sealed (multiple, fused)	Trough
4.00	Sealed (multiple, fused)	(Nothing)

Supplementary Table 3. Morphological statistics from the largest ("Cluster #1") and second-largest ("Cluster #2") lattice regions identified at the end of the simulations shown in Supplementary Fig. 3.

Switching rate (MD time steps)	Active proportion	Cluster #1	Cluster #2
1×10^4	50%	Troughs (multiple, fused)	(Nothing)
1×10^5	25%	Sealed	Sheet
1×10^5	50%	Sealed + trough	Trough
5×10^5	10%	Partial cone	(Nothing)
5×10^5	25%	Sealed (multiple, fused)	(Nothing)
5×10^5	50%	Trough	Sealed

Supplementary Table 4. Morphological statistics from the largest ("Cluster #1") and second-largest ("Cluster #2") lattice regions identified at the end of the simulations shown in Supplementary Fig. 4.

Supplementary Note 1 : CG model generation

We seek to generate our CG model based directly on known experimental data, influenced by the generation of CA cylinders in vitro [1-5] and the high-resolution hexameric [5] and pentameric [6] capsomers by the Yeager laboratory and colleagues. Extensive discussions with these authors inspired the incorporation of not only capsomer structural information but also the properties of CA cylinder geometry directly into CG model generation, with initial work performed using experimental cylinder data provided by the Yeager research group. We represent a CG capsid protein using three elements: the internal protein structure, the overall molecular shape via excluded volume effects, and finally the presence of specific protein/protein interfaces of known importance to capsid self-assembly. The fundamental CG capsid protein is thus a simple internal structural

framework onto which we add excluded volume information to reproduce the molecular shape of CA (as indicated by experimental data). Finally, a small number of specific attractive interactions are introduced, to represent known-important functional regions in CA assembly [7].

Parameterization of CG models of capsid protein using all-atom simulations via e.g., umbrella sampling or metadynamics is problematic. The conformational freedom of capsid protein is drastically reduced in mature lattice versus solution, and the near-fixed positions of CA in mature lattice regions ensure that inadequate sampling of phase space is almost guaranteed. Unfortunately, the properties of the capsid protein are sensitive to the specifics of the local environment: for example, the R18 side chains on helix 1 of the CA NTD point directly into the center of a pentameric or hexameric ring [5,6], resulting in several charged side chains in fairly close proximity. Attempts to calculate e.g., a potential of mean force curve (PMF) using two CA monomers will therefore fail to capture the properties of NTDs arranged into complete rings. Another example can be observed in the region of threefold lattice symmetry [8], where interactions within a clustered group of alpha helices from the CTDs of three different monomers suggests that producing the correct information requires all three monomers to be present. The specific NTD/CTD conformation of adjacent monomers is likewise dependent on the local curvature of the lattice, with exhaustive sampling of all possible lattice curvatures impractical. The parameterization of CG models of CA by the isolation of structural elements, followed by all-atom simulations with conventional free-energy approaches, is therefore fraught with problems due to non-local influences for various molecular properties, and the difficulties in defining adequate collective variables / reaction coordinates for enhanced sampling techniques.

In addition to the technical difficulties in CG CA model parameterization using all-atom simulations, there are indications that weak binding effects between proteins (such as displayed by HIV-1 CA) are not accurately captured in virion-relevant conditions by existing all-atom force fields [9]: if this is the case, then even if the CG model reproduced exactly the interactions revealed by the all-atom simulations, those interactions may not be a reliable description of the true system.

Internal structure of CG capsid protein

The CG model of HIV-1 CA treats the NTD and CTD as distinct local structures composed of the alpha helices in each domain. CG particles therefore correspond to Cα atoms in alpha helices. The CG NTD is generated from a superposition of the NTDs in PDB 3H4E [5], with average Cα positions used as the location of CG particles. An elastic network model (ENM) connects CG particles in an alpha helix to preserve the local helix structure with harmonic spring constant $K = 10$ kcal/mol/Å^2. To maintain the relative positions of alpha helices, each CG particle was connected to its closest neighbor from every other helix ($K = 10$ kcal/mol/Å^2).

The CG CTD is based on chain A of PDB 2KOD [8], with alpha helices extracted and connected as for the NTD. The CTDs of two CG monomers in the CA dimer are connected with an ENM based on PDB 2KOD ($K = 10$kcal/mol/Å^2). We note that reproduction of detailed structural properties (e.g., crystallographic beta factors etc) is not our intention, but rather we seek to efficiently reproduce the basic internal structure of CA.

In the natural CA protein, the NTD and CTD of a monomer are connected with a flexible linker region. As we require the controlled partitioning of CA into "assembly competent" and "assembly incompetent" structural populations, a fully flexible linker region is unsuitable. Instead, an additional weak ENM (K = 0.01 Kcal/mol/Å^2) connects the NTD and CTD to provide limited conformational flexibility. ENM resting distances were based on PDB 3H4E: as this describes isolated hexamers, additional basic testing was used to verify the approach can represent monomer structures suitable for mature lattice as described below.

Given the hexagonal spacing of mature CA lattice, mathematical approximations to hexagonal cylinders with tube radius comparable to electron density map EMD-5136 [8] were generated as decoy structures (e.g. Supplementary Fig. 1A) in order to investigate whether the "correct" experimental structure could be distinguished by a CG ENM model. CG hexamers were superposed onto the decoys, with adjacent NTD pairs in hexamers restrained by ENMs based on PDB 3H4E to preserve the hexameric NTD structures (K = 10 kcal/mol/Å^2). It is important to note that these restraints were used only in this analysis, and are not used in the final CG model. All decoy structures were energy minimised by Langevin dynamics, and stress in the weak intra-molecular NTD/CTD ENMs was measured. The decoy structure with lowest stress was compared to EMD-5136 using the Situs software, and was found to identify the appropriate tube morphology (Supplementary Fig. 1A). We thus conclude that a weak ENM connecting the NTD and CTD in a CG capsid protein monomer is an adequate structural approach for our purposes.

Excluded volume properties of CG capsid protein

CG particles have default excluded volume separation of 1 nm (ie CG particle radii are 5 Å). CG monomers were superposed onto PDB structures 3H4E and 3POA, with excluded volume separations adjusted for CG particle types < 1 nm apart. The same analysis was applied to CG monomers in the minimised decoy structure matching EMD-5136 (as described earlier), to identify additional steric clashes that were not present in isolated capsomers. This treatment produces a basic excluded volume model of CA to capture the fundamental CG molecular shape. Excluded volume interactions are a soft cosine potential:

$$U_{excl}(r_{ij}) = A[\,1 + \cos(\pi\, r_{ij} / r_{cut})\,],\; r_{ij} < r_{cut}$$

Here, r_{ij} is the separation of CG particle centers with r_{cut} the onset of excluded volume repulsion for specific particle pairs *i,j*. The value of A is 10 kcal mol^{-1} Å^{-1} in all cases.

We note the precise functional form of the excluded volume interactions are unimportant, provided the basic molecular shape is captured. This assessment is supported by our previous CG model of CA [10] where a different functional form was used, but the onset of repulsive forces were determined via similar analysis: this model also reproduced the steric packing of mature CA lattice.

Protein/protein self-assembly interfaces of the CG capsid protein

Mutation data [7] and experimental structures indicate three important protein/protein interfaces for capsid self-assembly: adjacent CA NTDs in ring structures [2], the NTD/CTD interface between adjacent CA monomers in a ring [5], and the CTD interface at the center of a threefold symmetry axis in mature lattice [8]. We initially attempted to model these interfaces with simple Lennard-Jones 12-6 interactions as in our previous CG model [10] but this approach failed to self-assemble without systematic defects in lattice structure, regardless of the interaction parameters. A more sophisticated approach was therefore required.

Given the difficulties in calculating, e.g., atomistic MD PMFs to describe nonbonded interactions for the capsid protein (as described previously) we consider instead the capsid lattice itself. The relative 3-D positions of CG particles in adjacent monomers might be expected to follow the central limit theorem for large numbers of samples over many potential lattice conformations, producing a normal distribution. It is therefore logical to approximate this property using a functional form such as a Gaussian potential. To estimate the mean value of the distribution, consecutive pairs of CA monomers *A,B* around NTD ring structures were identified from experimental hexamer (3H4E) and pentamer (3P0A) structures [5,6]. The monomer pairs were superposed onto a common reference frame via monomer *A*, and particle centroid coordinates are calculated from the set of *B* monomers. The centroid coordinates are therefore the estimated positions of CG particles in an adjacent monomer relative to an arbitrary reference monomer in mature lattice, with the root-mean-squared deviation (RMSD) of the centroid suggesting how well conserved the position might be.

Centroid analysis was performed using experimental structures from both hexamer (3H4E) and pentamer (3P0A) data sets, to examine well-conserved particle locations (as ranked by centroid RMSD) for NTD/NTD and NTD/CTD interfaces. For the NTD/NTD interface, particles on helix 3 ranked highly as conserved locations: the top-5 ranked particle locations from both the hexamer and pentamer data sets were all present on helix 3. Similar analysis was performed for centroids relevant to the NTD/CTD interface. Data on the particles selected to model these interfaces were as follows:

The threefold CTD interface was examined by extracting trimer-of-dimers structures from the minimised decoy structure, and extracting the centroid location of the CG particle for residue 204 (located on helix 10 of the CTD) from adjacent CTDs moving clockwise around the threefold symmetry axis.

The selected centroid locations were added to the ENM structures of the CG model as additional CG "ghost" particles to act as "binding locations" for specific CG particles of adjacent monomers in mature lattice (residues 51 and 57 to the NTD, residue 63 and 204 to the CTD, Supplementary Fig. 1B). These additional particles interact only with the specific CG particle type that was used to generate their positions, and are otherwise entirely inert.

The functional form of the binding potential is a double-Gaussian:

$$U_{bind}(r_{ij}) = -[\, A\times\exp(-B\, r_{ij}^2) + C\times\exp(-D\, r_{ij}^2)\,],\ r_{ij} < r_{cut}$$

Here, r_{ij} is the separation of CG particle centers with r_{cut} 20 Å. The parameters A and B describe the depth and width of a Gaussian energy well to control the close-ranged fine detail in a binding pocket, with C and D the depth and width of a longer ranged Gaussian to encourage aggregation. The double-Gaussian approach was found to be necessary from extensive trial simulations: the absence of the long-ranged component resulted in no observable assembly, and significant lattice defects occurred in the absence of the sort-ranged component. For simplicity, we use symmetrical parameters in the attractive interactions: A = 1 kcal mol^{-1}, B = 0.1 Å^{-2}, C = 2 kcal mol^{-1}, D = 0.01 Å^{-2} for all interfacial binding pockets, determined from extensive test simulations under various conditions, to allow the study of self-assembly in CG MD simulations with CA concentrations and molecular crowding levels relevant to the HIV-1 virion.

Inert CG molecular crowding agent

Guided by the studies of del Alamo et al. [11], we use a simple inert molecular crowding agent to approximate the mass and excluded volume of Ficoll 70. The molecular structure of Ficoll 70 appears to be intermediate between globular and linear [12], with Stokes radius estimated as ≈ 5.1 nm and average molecular mass 70 kDa (Amersham Biosciences product specification sheet). One "instance" of Ficoll 70 is therefore modeled using two spherical ENMs, each consisting of 42 CG particles with excluded volume radii to produce an overall sphere diameter ≈ 5.1 nm (Supplementary Fig. 1C) and relative mass 35 kDa. To minimise any potential effects from complicated crowder geometries on CA self-assembly, we allow the two spheres to move independently to approximate the excluded volume of specific Ficoll 70 densities using very simple molecular shapes. Excluded volume interactions use the soft repulsive potential described previously. We note that the combination of two smaller spheres and soft repulsion may underestimate the true occluded volume of a specific density of Ficoll 70, and hence the resultant crowding effects.

CG molecular masses

Increased molecular crowding enhances capsid protein self-assembly [11], and so the nucleation and growth of CA lattice is not diffusion limited [13]. Initial test simulations with full mass assigned to the CG capsid protein (≈ 25 kDa) and inert crowding agent (≈ 2 x 35 kDa) indicated very long simulation times could be required to study CA self-assembly. In order to provide access to even longer effective time scales in the CG simulations, improving the detection of nucleation and growth under the widest possible conditions, we therefore reduced the effective mass of each CG particle in the capsid protein to 10 Da (≈ 20-fold reduction) with the mass of CG crowding agent rescaled as appropriate to the new CA mass. It should be noted that in classical statistical mechanics the free energy landscape is not dependent on the mass of the particles. Moreover, in terms of the dynamics of the self assembly, the CG timescale is not the same as the physical timescale and must therefore be calibrated independently in the absence of an explicit dynamical correction term included in the equations of motion (the latter being difficult to precisely formulate theoretically and to implement algorithmically). Uniformly changing the mass of the CG particles merely affects and adjusts the timescale calibration factor, but it is not likely to change the overall sequence of assembly events.

Supplementary Note 2 : Structural motif counts as a function of simulation time.

Here, the green line (indicating the total number of hexamers identified in the system as a function of simulation time) can be considered a general proxy for the level of mature-style lattice assembly present in the system. The total level of "CA assembly" in can therefore be inferred from this line.

Of the six simulations in Supplementary Fig. 2 where capsid lattice nucleated and grew, morphological analysis was performed on the largest and second-largest CA structure present at the end of the simulation (see also Fig. 2 of the main manuscript). Here, we define a cluster to be a capsid lattice region containing at least one hexamer or pentamer structure. The results were categorized into three basic morphologies: sheets (flat or curving lattice regions), troughs (sheets where one or more of the ends of the sheet have joined together), and sealed (structures with closed or almost-closed surfaces, e.g. pill-shaped or cone-shaped).

In the case of CA = 4 mM and 200 mg/mL crowding, several pill shaped structures appear to have fused into a single amorphous structure, leaving no other lattice assemblies regions significantly larger than a trimer-of-dimers. Across the simulations listed in Supplementary Table 2: 3/6 contained sheets, 2/6 contained troughs, and 4/6 contained sealed structures (akin to small, malformed capsids).

Supplementary Note 3 : Structural motif counts as a function of simulation time under fixed levels of molecular crowding

Here, the green line (indicating the total number of hexamers identified in the system as a function of simulation time) can be considered a proxy for the general level of mature-style lattice assembly present in the system. The total level of "CA assembly" in can therefore be inferred from this line.

Of the 14 simulations in Supplementary Fig. 3 where capsid lattice nucleated and grew, morphological analysis was performed on the largest and second-largest CA structure present at the end of the simulation (see also Fig. 3 of the main manuscript). Analysis follows the approach described in Supplementary Note 2.

In several cases, multiple structures appear to have fused into a single amorphous assembly, leaving no other lattice assemblies regions significantly larger than a trimer-of-

dimers. There are also occasions where sealed structures appear to have merged with trough-like structures at higher $[CA]_+$ ("Sealed + trough"). Across the simulations listed in Supplementary Table 3: 5/14 contained sheets, 8/14 contained troughs, and 8/14 contained sealed structures (akin to small, malformed capsids).

Supplementary Note 4 : Structural motif counts as a function of simulation time under fixed levels of molecular crowding with different active/inactive CA conformational switching intervals

Note: CG-MD simulation time scales represent longer effective "real world" times: indeed, this is an important advantage of CG models in the study of self-assembly phenomena over time scales inaccessible to more detailed atomistic MD approaches. The switching rates studied (1×10^4, 1×10^5, and 5×10^5 CGMD time steps, corresponding to 0.1 ns, 1 ns, and 5 ns of CG time respectively with CGMD time step of 10 fs) are therefore for qualitative comparison of the effects of population switching, rather than a precise quantitative measure that corresponds to real-world timescales.

Here, the green line (indicating the total number of hexamers identified in the system as a function of simulation time) can be considered a general proxy for the level of mature-style lattice assembly present in the system. The total level of "CA assembly" in can therefore be inferred from this line.

Of the 6 simulations in Supplementary Fig. 4 where capsid lattice nucleated and grew, morphological analysis was performed on the largest and second-largest CA structure present at the end of the simulation (see also Fig. 6 of the main manuscript). Analysis follows the approach described in Supplementary Note 2.

In several cases, multiple assembling structures appear to fuse, leaving no other lattice assemblies regions significantly larger than a trimer-of-dimers. There are also occasions where sealed structures appear to have merged with trough-like structures ("Sealed + trough"). Across the simulations listed in Supplementary Table 4: 1/6 contained sheets, 4/6 contained troughs, and 3/6 contained sealed structures (akin to small, malformed capsids).

Supplementary Note 5 : Additional independent simulation of the system described in Fig. 6 of the main manuscript.

A time-series plot of structures from a repeat simulation for the system shown in Fig. 6 of the main manuscript is shown in Supplementary Fig. 5. This simulation was started with a different initial momentum distribution, but identical starting coordinates.

Note: There are indications that the "shoulder" visible on the upper left region of the truncated cone shown in Supplementary Fig. 5 is formed after self-obstruction of the growing lattice structure across periodic boundaries.

Supplementary Note 6 : Deviations from expected CTD/CTD dimer interface conformations in all-atom MD simulations of the HIV-1 viral capsid

Supplementary Fig. 6 presents example CTD/CTD dimer configurations from atomistic MD simulation of a model capsid[14] structure compared to the CTD/CTD dimer structure from PDB accession code 2KOD[8]. **Source data:** http://www.ks.uiuc.edu/Research/hiv/

Supplemental References

1 Ganser, B. K., Li, S., Klishko, V. Y., Finch, J. T. & Sundquist, W. I. Assembly and analysis of conical models for the HIV-1 core. *Science* **283**, 80-83 (1999).

2 Ganser-Pornillos, B. K., Cheng, A. & Yeager, M. Structure of full-length HIV-1 CA: a model for the mature capsid lattice. *Cell* **131**, 70-79 (2007).

3 Ganser-Pornillos, B. K., von Schwedler, U. K., Stray, K. M., Aiken, C. & Sundquist, W. I. Assembly properties of the human immunodeficiency virus type 1 CA protein. *J. Virol.* **78**, 2545-2552 (2004).

4 Pornillos, O., Ganser-Pornillos, B. K., Banumathi, S., Hua, Y. & Yeager, M. Disulfide bond stabilization of the hexameric capsomer of human immunodeficiency virus. *J. Mol. Biol.* **401**, 985-995 (2010).

5 Pornillos, O. *et al.* X-ray structures of the hexameric building block of the HIV capsid. *Cell* **137**, 1282-1292 (2009).

6 Pornillos, O., Ganser-Pornillos, B. K. & Yeager, M. Atomic-level modelling of the HIV capsid. *Nature* **469**, 424-427 (2011).

7 von Schwedler, U. K., Stray, K. M., Garrus, J. E. & Sundquist, W. I. Functional surfaces of the human immunodeficiency virus type 1 capsid protein. *J. Virol.* **77**, 5439-5450 (2003).

8 Byeon, I. J. *et al.* Structural convergence between Cryo-EM and NMR reveals intersubunit interactions critical for HIV-1 capsid function. *Cell* **139**, 780-790 (2009).

9 Petrov, D. & Zagrovic, B. Are current atomistic force fields accurate enough to study proteins in crowded environments? *PLoS Comput. Biol.* **10**, e1003638 (2014).

10 Grime, J. M. & Voth, G. A. Early stages of the HIV-1 capsid protein lattice formation. *Biophys. J.* **103**, 1774-1783 (2012).

11 del Alamo, M., Rivas, G. & Mateu, M. G. Effect of macromolecular crowding agents on human immunodeficiency virus type 1 capsid protein assembly in vitro. *J. Virol.* **79**, 14271-14281 (2005).

12 Fissell, W. H. *et al.* Ficoll is not a rigid sphere. *Am. J. Physiol. Renal Physiol.* **293**, F1209-1213 (2007).

13 Minton, A. P. Influence of macromolecular crowding upon the stability and state of association of proteins: predictions and observations. *J. Pharm. Sci.* **94**, 1668-1675 (2005).

14 Zhao, G. *et al.* Mature HIV-1 capsid structure by cryo-electron microscopy and all-atom molecular dynamics. *Nature* **497**, 643-646 (2013).
